# Insights Into the Effects of Mucosal Epithelial and Innate Immune Dysfunction in Older People on Host Interactions With *Streptococcus pneumoniae*

**DOI:** 10.3389/fcimb.2021.651474

**Published:** 2021-05-25

**Authors:** Caroline M. Weight, Simon P. Jochems, Hugh Adler, Daniela M. Ferreira, Jeremy S. Brown, Robert S. Heyderman

**Affiliations:** ^1^ Research Department of Infection, Division of Infection and Immunity, University College London, London, United Kingdom; ^2^ Department of Parasitology, Leiden University Medical Center, Leiden, Netherlands; ^3^ Department of Clinical Sciences, Liverpool School of Tropical Medicine, Liverpool, United Kingdom; ^4^ Tropical and Infectious Diseases Unit, Liverpool University Hospitals National Health Service (NHS) Foundation Trust, Liverpool, United Kingdom; ^5^ Respiratory Medicine, University College London, London, United Kingdom

**Keywords:** epithelium, pneumococcus (*Streptococcus pneumoniae*), innate immunity, inflammageing, older individuals

## Abstract

In humans, nasopharyngeal carriage of *Streptococcus pneumoniae* is common and although primarily asymptomatic, is a pre-requisite for pneumonia and invasive pneumococcal disease (IPD). Together, these kill over 500,000 people over the age of 70 years worldwide every year. Pneumococcal conjugate vaccines have been largely successful in reducing IPD in young children and have had considerable indirect impact in protection of older people in industrialized country settings (herd immunity). However, serotype replacement continues to threaten vulnerable populations, particularly older people in whom direct vaccine efficacy is reduced. The early control of pneumococcal colonization at the mucosal surface is mediated through a complex array of epithelial and innate immune cell interactions. Older people often display a state of chronic inflammation, which is associated with an increased mortality risk and has been termed ‘Inflammageing’. In this review, we discuss the contribution of an altered microbiome, the impact of inflammageing on human epithelial and innate immunity to *S. pneumoniae*, and how the resulting dysregulation may affect the outcome of pneumococcal infection in older individuals. We describe the impact of the pneumococcal vaccine and highlight potential research approaches which may improve our understanding of respiratory mucosal immunity during pneumococcal colonization in older individuals.

## Introduction

William Osler, a Canadian physician, who himself died of pneumonia, wrote in his book *The Principles and Practice of Medicine*: “In the aged, the chances are against recovery. So fatal that it has been termed the natural end of the old man” (Osler, 1892).

Much has changed since, with a huge global public health effort to reduce the burden of pneumonia and invasive pneumococcal disease (IPD), particularly in young children. However, the 1.5 billion people worldwide who are >65yrs (older individuals) now outnumber those <5yrs and, by 2050, will outnumber those aged 15–24yrs when there is predicted to be 426 million people >80yrs (U.N, 2019). Community Acquired Pneumonia (CAP) is common in older individuals, particularly men, with infection by *Streptococcus pneumoniae* as the leading cause (Kaplan et al., 2002; Janssens and Krause, 2004; Stupka et al., 2009). In 2016, pneumococcal pneumonia was responsible for ~494,340 deaths globally in individuals >70yrs (Collaborators, 2018).

Why older people are so vulnerable to disease caused by *S. pneumoniae* is likely to be multifactorial including co-morbidities, relative immunodeficiency, malnutrition and defective swallowing (Janssens and Krause, 2004; Zalacain, 2004; Arndt, 2015). Disease follows pneumococcal carriage and reported nasopharyngeal and oropharyngeal carriage rates in older people vary between 0–39% (Krone et al., 2015; Adler et al., 2020; Almeida et al., 2020; Smith et al., 2020; Yasuda et al., 2020). Unlike adults aged 18–64yrs, older adults do not appear to benefit from the natural immune effects of pneumococcal colonization events that are thought to protect against re-colonization and disease (Ferreira et al., 2013; Adler et al., 2020). Older people often display a disorganised inflammatory state, which is associated with an increased mortality risk and has been termed ‘Inflammageing’ (Franceschi and Campisi, 2014; Ferrucci and Fabbri, 2018). This may compromise upper respiratory mucosal immunity, mediated by the nasopharyngeal epithelium and other cellular and soluble innate immune components (Simell et al., 2012; Wilson et al., 2015; Jochems et al., 2018; Jochems et al., 2019a; Weight et al., 2019).

**Figure 1:**
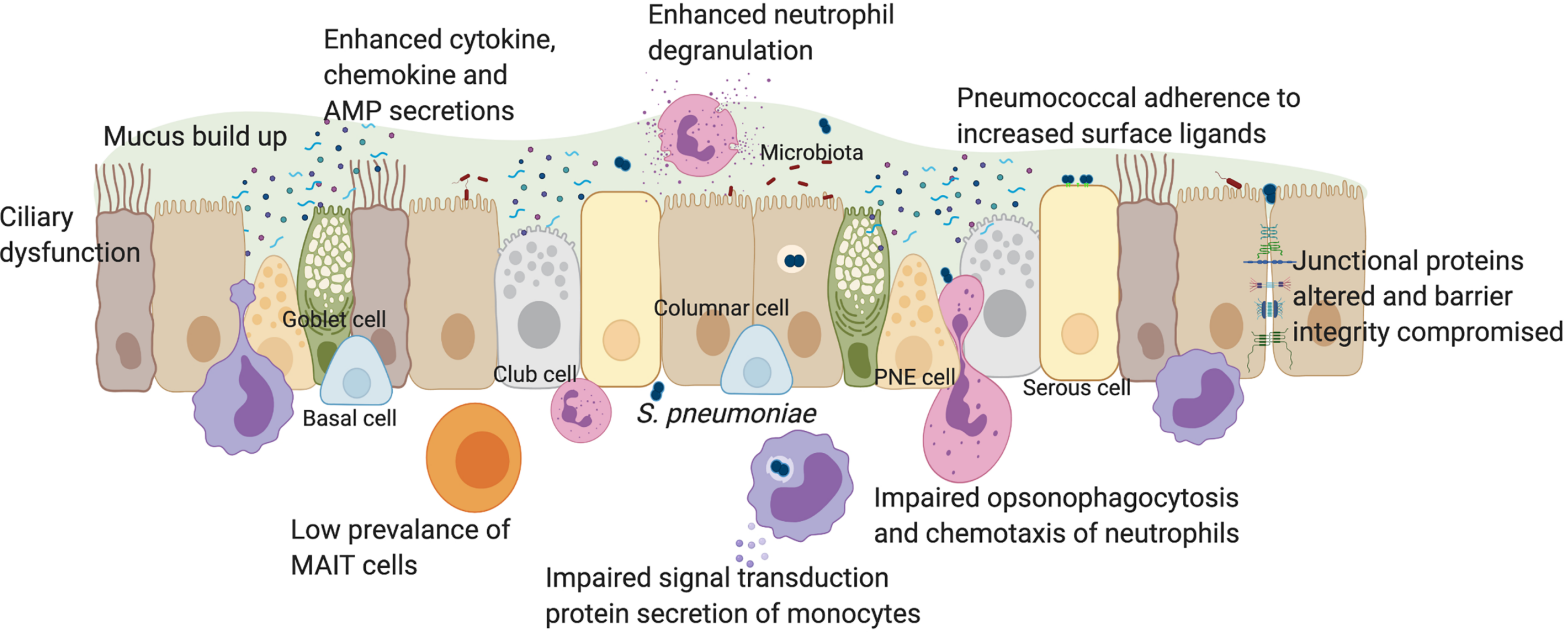
The impact of pneumococcal infection on mucosal immunity in older individuals. The human respiratory epithelium includes many cell types all of which contribute to the development of innate immunity through a physical barrier held together by junctional proteins, and a chemical barrier *via* secretions of mucus, cytokines, chemokines, antimicrobial peptides, vitamin D and retinoic acid. Innate immune cells such as monocytes, neutrophils and MAIT cells are also present in the mucosa. In older individuals, there is a loss of physical movement both at a mechanical elasticity level and a lack of cilia beating which impacts on mucus clearance. This contributes towards increased prevalence of luminal factors such as cell debris, secreted factors, microbiota and pathogens such as *S. pneumoniae* which trigger already elevated baseline levels of cytokines such as IL-6, IL-8 and TNFα. In younger adults, epithelial-derived secretion of anti-microbial peptides such as cathelicidin and NFκB activation, leads to autophagy. Impaired autophagy and type 1 interferon responses in older individuals may lead to suppressed IFNβ levels, increasing pneumococcal load. Increased expression of epithelial senescence markers and pneumococcal ligands such as PAFr in older people enhances pneumococcal colonization, influencing adhesion, micro-invasion and transmigration potential. Vitamin D deficiency in older people may affect epithelial barrier integrity. In younger adults, disruption to barrier function after pneumococcal infection affected the expression of junctional proteins such as Claudins. In older adults, dysregulation of barrier may enhance rates of pneumococcal transmigration, infiltration of innate immune cells and inflammation. Although MAIT cells are rare in the airway of older individuals, neutrophil prevalence is enhanced, which elevates degranulation and reactive oxygen species levels following pneumococcal infection. However, neutrophil ability for opsonophagocytosis and chemotaxis is impaired in older individuals. Monocyte function may also be impaired in signal transduction and secrete less IL-6, IL-8 and TNFα during infection, in comparison to younger adults. PNE cell, pulmonary neuroendocrine cell; AMP, anti-microbial peptides. Created with Biorender.com.

In this review we discuss the impact of inflammageing on innate immunity to *S. pneumoniae* in older people, summarised in **Figure 1**. We outline the impacts of the pneumococcal vaccine in older individuals and experimental approaches which may lead to deeper understanding of how the pneumococcus affects this vulnerable population.

## Physiology and Ageing

### Pulmonary Physiology

In humans, there is a decrease in pulmonary elasticity, loss of respiratory muscular strength, decreased ciliary beating and mucus velocity with age (Ho et al., 2001; Janssens and Krause, 2004). These changes occur from a combination of genetic predisposition, inflammageing and environmental exposure, which involve a wide range of molecular and cellular changes and impairment of cell-cell communications (Brandenberger and Muhlfeld, 2017).

### Microbiome

It is becoming increasingly apparent that the microbiome is an important determinant of lung and gut homeostasis and the development of disease, particularly in older individuals (de Steenhuijsen Piters et al., 2016; Schenck et al., 2016; Man et al., 2017; Ragonnaud and Biragyn, 2021). It remains uncertain whether changes in the composition and diversity of microbiota represent a cause or consequence of pneumonia (de Steenhuijsen Piters et al., 2016). Older individuals with pneumonia exhibit increased abundance of species such as *S. pneumoniae*, Rothia and Lactobacilli, but decreased overall anaerobic bacterial diversity in the upper respiratory tract (URT) (de Steenhuijsen Piters et al., 2016). In a human experimental pneumococcal challenge model (EHPC), low density pneumococcal carriage was associated with a stable mucosal microbiome (baseline presence of Corynebacterium/Dolosigranulum species) and a less pro-inflammatory phenotype (de Steenhuijsen Piters et al., 2019). Whether different pneumococcal strains interact differently with the respiratory microbiome during colonization (Cremers et al., 2014) and how this affects older individuals, remains to be determined. The role of intestinal microbiota on lung susceptibility to pneumococcal infection also warrants further investigation in humans as murine studies suggest that Nod-stimulating microbiota in the gut induce GMCSF-dependent immunity, which influences alveolar macrophage function during pneumococcal infection (Schuijt et al., 2016; Brown et al., 2017).

### Inflammageing

An imbalance of cytokine expression in older individuals is referred to as “inflammageing”, where damage to the tissue, changes in composition of the microbiome and cellular and immune senescence, all contribute to this state of chronic inflammation (Franceschi and Campisi, 2014). The contributors to inflammageing may include microbial translocation, chronic infections, mitochondrial dysfunction and accumulation of DNA damage (Fulop et al., 2017; Ferrucci and Fabbri, 2018). This increased inflammation extends to the lungs, as healthy individuals >65yrs have elevated levels of IL-6, IL-8 and higher numbers of neutrophils in bronchoalveolar lavage (BAL) samples (Thompson et al., 1992; Meyer et al., 1996). The increased susceptibility to respiratory tract infections has also been linked to the heightened inflammatory status in older people. In a large prospective study of people aged 70-79yrs, being in the highest tertile for systemic IL-6 and TNF levels was associated with 1.6-1.7-fold increased risk for developing CAP (Yende et al., 2005). Inflammation, in particular IL-6 levels, at time of admission to hospital is also associated with CAP disease severity (Glynn et al., 1999; Antunes et al., 2002). Whether this represents more severe disease, or a pre-existing heightened inflammatory state is uncertain. For example, at admission, in a cohort of 22 patients with pneumonia, of which 19 had confirmed *S. pneumoniae*, patients <55yrs had increased levels of IL-6 compared to patients >68yrs (Bruunsgaard et al., 1999). However, at 7 days post admission, pro-inflammatory cytokines TNF and soluble TNF receptor I remained elevated in older adults, while they had returned to baseline in young adults. This increased inflammatory state with age may therefore contribute to dysregulation of immunomodulation of innate immune cells such as neutrophils, cytokines and chemokines (Williams et al., 2015) and ultimately, pneumococcal colonization, increasing the chance of IPD in older individuals.

## Epithelial Cell Function and Ageing

The nasopharyngeal epithelium provides the first line of defense against respiratory pathogens. An intact physical barrier, together with epithelial secretions of mucus, anti-microbial peptides and proteins, chemokines and cytokines, forms the basis of epithelial-derived innate immunity (**Figure 1**). Hence, age related alterations in epithelial responses would have profound effects on pneumococcal colonization, as summarized in **Table 1**.

**Table 1:** Epithelial cell changes and ageing in the context of pneumococcal infection.

Molecular changes	Pneumococcal outcome	Impact on epithelial barrier
↑Keratin 10, laminin receptor, PAFr expression	↑ Pneumococcal adherence, micro-invasion and toxin concentrations	↑ Epithelial damage, inflammation and immune cell recruitment
↓ Claudin-5, -7, -10, Occludin, ZO-1, VE-cadherin expression	↑ Pneumococcal transmigration across the epithelial barrier	↑ Barrier permeability, NFκB activation, inflammation and immune cell recruitment
↑Claudin 2 expression		↓ Transepithelial electrical resistance
↓ Vitamin D signalling		↑ Epithelial damage, inflammation and immune cell recruitment
↓ LL-37, β defensin -2, -3, -4, S100A7, -8, -9, Lipocalin and RNase 7 secretion	↑ Pneumococcal load and toxin concentrations	↓Autophagy, NLRP3 inflammasome activation
↑ Or ↓ IL-6 production	↓ Or ↑ Effects on pneumococcal-epithelial associations, micro-invasion and transmigration	↓ Or ↑ Affecting barrier permeability, proliferation and epithelial repair

### Epithelial Cell Activation

*S. pneumoniae* binds to a variety of epithelial receptors including Keratin 10, laminin receptor and platelet-activating factor receptor (PAFr), the expression of which is altered in older individuals, thus potentially influencing pneumococcal adherence and susceptibility towards disease. For example, levels of Keratin 10, laminin receptor and PAFr are elevated in aged mice, human lung tissue and senescent A549 cells (Hinojosa et al., 2009; Shivshankar et al., 2011) which could contribute to altered outcomes after pneumococcal colonization. Pneumococcal micro-invasion of the epithelium *in vitro* is also associated with epithelial secretion of cytokines and chemokines and a transcriptomic enrichment of innate signaling pathways including Toll receptor cascades, NFκB and MAPK activation (Weight et al., 2019). In the EHPC model, an epithelial transcriptomic signature that was associated with bacterial clearance has been identified, indicating the involvement of epithelial activation in the control of pneumococcal colonization (Weight et al., 2019). Furthermore, *in vitro* epithelial cell models reveal that activation of p65, upregulation of the histone demethylase KDM6B and IL-11 secretion are associated with protection from epithelial damage following pneumococcal infection (Connor et al., 2020). We therefore speculate that in older adults, where pneumococcal micro-invasion and cellular senescence may be enhanced, dysregulation of these pathways and regulatory mechanisms could exacerbate invasive disease.

*Mucosal Barrier Function:* The expression profiles of intercellular junctional proteins such as Claudins, ZO-1 and E-Cadherin, determine the permissiveness of the respiratory epithelium to the passage of microbes across the barrier. Changes in epithelial junctional expression during pneumococcal infection leads to structural reorganization of the barrier. For example, *in vitro* infection of human nasopharyngeal cells with *S. pneumoniae* is initially associated with decreased permeability to 4kDa FITC-dextran, indicating a strengthened barrier (Weight et al., 2019). However, over time (>8 hours post-infection, when bacterial replication and autolysis have also occurred) barrier integrity is altered, demonstrated through decreased expression of Occludin, ZO-1, Claudin-5 and VE-cadherin in the alveoli epithelium and endothelium in younger adults lung explants (Peter et al., 2017). Furthermore, downregulation of Claudin-7 and Claudin-10 in human and murine epithelial cells, led to increased pneumococcal transmigration across the epithelial barrier (Clarke et al., 2011).

The vitamin D receptor (VDR) regulates epithelial barrier function (Chen et al., 2018). In *Salmonella* infected VDR^-/-^ mice, Claudin 2 upregulation was associated with leaky intestinal barrier, increased pathology and upregulation of NFκB (Zhang et al., 2019), a critical regulator of inflammageing (Salminen et al., 2008) and tight junction protein expression (Ward et al., 2015). In the older human intestine, Claudin-2 upregulation has been detected, which was accompanied by decreased transepithelial electrical resistance and increased permeability (Man et al., 2015). Activation of NFκB following pneumococcal infection is widely reported (Malley et al., 2003; Weight et al., 2019), and Vitamin D deficiency is more severe in older generations (Hirani and Primatesta, 2005; Jolliffe et al., 2013), and there is evidence to suggest that supplementation of Vitamin D could be beneficial in boosting immunity and reducing acute respiratory infections (Martineau et al., 2017; Chambers et al., 2021). Whether regulation and junctional protein responses in the URT are altered with age in humans, and how this contributes to control of pneumococcal colonization, remains to be determined.

### Antimicrobial Peptides and Proteins (AMPs)

An important factor of epithelial innate immunity includes AMPs that neutralize toxins and eliminate pathogens (Hiemstra, 2001). Infection of human corneal epithelial cells with pneumococcus induced NFκB activation leading to the secretion of LL-37, β defensin -2, -3, -4, S100A7, S100A8, S100A9, Lipocalin and RNase 7 (Sharma et al., 2019). LL-37 plays a role in wound healing, can induce autophagy in a 1,25-dihydroxyvitamin D3 dependent manner, can activate the NLRP3 inflammasome in a model of *P. aeruginosa* and, is bactericidal against *S. pneumoniae* and *Mycobacterium tuberculosis* (Nijnik and Hancock, 2009; Yuk et al., 2009; McHugh et al., 2019; Sharma et al., 2019). Older individuals maintain similar levels of baseline production of cathelicidins and β defensin 2 in serum compared to younger adults (Castaneda-Delgado et al., 2013). However, in aged mice, CRAMP expression, the murine homolog of LL-37, was not upregulated following pneumococcal infection, compared to younger adults (Krone et al., 2013). This suggests a potential dysregulation of AMPs in older individuals and the implications for the control of *S. pneumoniae* at the mucosal surface warrants further investigation.

### Cytokines and Chemokines

AMPs also induce the secretion of cytokines and chemokines like IL-6 and IL-8 from nasal epithelial cells, in an NFκB dependent manner (Pistolic et al., 2009). One might predict that given elevated levels of cytokines such as IL-6 and TNFα in older individuals (Yende et al., 2005; Man et al., 2015), epithelial cell responses may also differ in the response to pneumococcal carriage. IL-6 is a pleiotropic cytokine and so elevated baseline secretion in older individuals may either enhance or weaken barrier integrity upon pathogenic challenge. For example, IL-6 regulates the expression of tight junction proteins such as Claudin 2 and increases intestinal barrier permeability (Suzuki et al., 2011; Man et al., 2015). This could also occur in the respiratory setting, which may increase pneumococcal transmigration across the epithelial barrier. Alternatively, IL-6 is also known to confer epithelial repair and promote proliferation (Kuhn et al., 2014), which may inhibit pneumococcal adherence to the epithelium. For example, co-infection with Influenza A increases susceptibility to *S. pneumoniae* in both adult and older mice and in younger adults, characterized by increased bacterial burden in the URT (Mina et al., 2014; Jochems et al., 2018; Gou et al., 2019). In the murine study, IL-6 production was required to maintain barrier function and macrophage phagocytic function, which played a role in pneumococcal control and clearance (Gou et al., 2019). Although secreted by infected human nasopharyngeal cells *in vitro* (Weight et al., 2019), levels of epithelial IL-6 secretion *in vivo* have not been directly investigated in adults or older individuals.

## Innate Immunity and Ageing

The degree of inflammageing likely influences the functional responses of monocytes/macrophages, neutrophils and *Mucosal-associated invariant T (*MAIT) cells to *S. pneumoniae*, which in turn, may be detrimental in controlling the outcome of nasopharyngeal pneumococcal colonization in older people, as summarized in **Table 2**.

**Table 2:** Changes in Innate Immunity with age.

Changes with age	Monocytes/Macrophage	Neutrophils	MAIT cells
Prevalence	↑ Alveolar macrophages	↑ Nose and lungs	↓ Blood
	↑ CD14++CD16+ monocytes		↓Total CD8+ T cells in nasal mucosa
Cellular changes	↓ TLR1/2/4	↑ CD11b	↑ Clonal expansion
	↓STING/TBK1/IRF3	↓Extracellular traps, migration and opsonophagocytosis	
	↓ Inflammasome activation		
Cytokine responses	↑ TNF baseline	↑ ROS, Proteinase	
	↓TNF, IL-6, IL-1β, IL-8, IFNβ		

### Monocytes and Macrophages

TLR1 levels are reduced on monocytes from older adults and TLR1/2 specific stimulation using Pam2SCK4 is associated with reduced responses in monocytes from older people (van Duin et al., 2007). Indeed, cytokine responses to pneumococcal or relevant ligands also appear decreased with age. Frail older individuals have increased baseline production of TNF by intermediate monocytes in particular, but show an impaired induction upon TLR1/2 or TLR4 stimulation (Hearps et al., 2012; Verschoor et al., 2014b). Upon heat-killed pneumococcal stimulation however, monocyte-derived macrophages in frail older people produce less TNF, IL-6, IL-1β and IL-8 and have a reduced capacity to kill *S. pneumoniae*. This is possibly related to defective PI3K-AKT signaling (Verschoor et al., 2014a), and/or insufficient activation of the NLRP3 inflammasome, as demonstrated in bone marrow derived macrophages from aged mice (Cho et al., 2018).

In younger adults, infiltration of classical monocytes into the nasal mucosa in the EHPC model coincides with initiation of pneumococcal clearance, while nasal myeloid cytokines correlate with clearance of colonization (Jochems et al., 2018). The impact of the respiratory monocyte/macrophage dysfunction described above on pneumococcal control in older people is not fully understood. However, altered monocyte subsets are an important contributor to a reduced ability to prevent pneumococcal infection in older people (Puchta et al., 2016). For example, reduced cytokine production to TLR1/2 agonists seem to be mediated by changes in CD14++ CD16+ intermediate and CD14+ CD16+ non-classical monocytes (Nyugen et al., 2010). In addition, alveolar macrophage numbers are higher in BAL samples from healthy older adults compared to younger adults (Thompson et al., 1992; Meyer et al., 1996), although how this affects innate immune responses to *S. pneumoniae* is unknown.

Murine studies have also identified age-related functional differences in monocyte/macrophage interactions with *S. pneumoniae* that may be relevant for disease pathogenesis. Puchta et al. demonstrated that TNF is a crucial mediator of the susceptibility to pneumococcal infections in inflammageing, as well as the alterations in monocyte subsets (Puchta et al., 2016). Increasing TNF with age led to premature egress of pro-inflammatory monocytes from bone marrow and increased levels of intermediate CD14++ CD16+ monocytes. Specific depletion of these monocytes or reduction in TNF levels enhanced immunity to pneumococcal infection and increased clearance in old mice (Puchta et al., 2016). Koppe found that following sensing of pneumococcal dsDNA by murine macrophages, STING (“stimulator of IFN genes”) binds to TBK1 (TANK-binding Kinase 1), leading to IRF3 (Interferon Regulatory Transcription Factor 3) activation and production of IFNβ, which assists pneumococcal clearance (Koppe et al., 2012). In aged mice, there is less STING/TBK1/IRF3 mRNA and protein expression compared to young mice infected with *S. pneumoniae* (Mitzel et al., 2014). This was associated with lower levels of IFNβ and higher bacterial burden in the lung, thought to be due to age-associated stress of the endoplasmic reticulum, resulting in increased autophagy-related protein 9a-STING complex formation (Mitzel et al., 2014), preventing STING complex formation with TBK1 (Saitoh et al., 2009).

### Neutrophils

There is a large body of evidence from both murine and human studies showing the importance of neutrophils in protecting against pneumococcal colonization of the nasopharynx (Lu et al., 2008; Weinberger et al., 2009; Jochems et al., 2018; Nikolaou et al., 2018). Impaired functional responses of neutrophils in older individuals may therefore also be detrimental during pneumococcal infection.

In the nose and lungs of healthy older people, neutrophils are highly abundant (Thompson et al., 1992; Meyer et al., 1996; Reiné et al., 2019). Excessive neutrophil recruitment to the lung following infection can mediate tissue damage and may exacerbate inflammation in older people (Menter et al., 2014). In frail older individuals, neutrophils have an immature profile with increased levels of intracellular reactive oxygen species (ROS) and cell surface expression of proinflammatory markers like CD11b (Verschoor et al., 2015). They have an impaired capacity to produce neutrophil extracellular traps (Hazeldine et al., 2014) and altered chemotaxis responses to respiratory infection, which leads to prolonged production of proteinase and a pro-inflammatory milieu (Sapey et al., 2014; Sapey et al., 2017). Neutrophils in older individuals may also be impaired in their opsonophagocytotic ability to several bacterial pathogens including *S. pneumoniae* and *S. aureus* (Wenisch et al., 2000; Butcher et al., 2001; Simell et al., 2011). Interestingly, vitamin E supplementation in aged mice prevented neutrophil migration and mortality during pneumococcal pneumonia (Ghanem et al., 2015), suggesting that regulating neutrophil activity in older individuals could be beneficial to the host.

### MAIT cells

Unconventional, innate-like T cells called MAIT cells, play important roles in the defense against bacterial and viral infections. Recently, studies with the EHPC model demonstrated that blood and nasal MAIT cells were associated with protection from pneumococcal colonization (Jochems et al., 2019b). They can be activated by conserved bacterial ligands derived from vitamin B (riboflavin) biosynthesis or indirectly *via* cytokines (Godfrey et al., 2019; Toubal et al., 2019). MAIT cells recognize precursors of the riboflavin synthesis pathway after presentation *via* MHC class I–related protein 1 (MR-1). This pathway is highly conserved in the pneumococcal genome, and MAIT cells can respond to pneumococcal isolates in both MR-1 dependent and independent manners (Kurioka et al., 2018). MAIT cells are depleted from blood in old age, with levels progressively dropping from 3-5% of T cells in young adulthood to below 1% in older adults (Novak et al., 2014; Walker et al., 2014; Loh et al., 2020). Remaining MAIT cells in this population show clonal expansion, similar to conventional CD8 T cells, and increased basal inflammation, although they retain potent anti-microbial function (Loh et al., 2020). There appears to be a substantial depletion of total CD8+ T cells from the nasal mucosa in older adults (Reiné et al., 2019) and the ratio of CD4/CD8 T cells increases in the lung in middle age (Meyer et al., 1996). Therefore, as MAIT cells are predominantly found within the CD8+ T cell compartment, we postulate that there is loss of MAIT cells at the mucosal surface in older individuals which negatively impacts on the control of pneumococcal colonization. This warrants further investigation.

### Th17 and T Regulatory Cells (Tregs)

In murine models, a Th17-mediated recruitment of monocytes and neutrophils leads to clearance of *S. pneumoniae* colonization (Lu et al., 2008; Zhang et al., 2009). Some human studies also suggest a role for Th17 cells in protection against colonization showing increased ratios of pneumococcal-specific Th17/Tregs with increasing age as carriage rates decrease (Mubarak et al., 2016) and a SNP in the IL17A gene associated with ~2-fold increased risk of pneumococcal colonization in children in the first year of life (Vuononvirta et al., 2015). However, a protective role for Th17 cells in humans has not been fully substantiated. We have shown acquisition of pneumococcal antigen-specific tonsillar Th1 T cells but not Th17 cells with age (Pido-Lopez et al., 2011). In the EHPC model, Th17 cells were found in the lung after colonization, which associated with increased bacterial killing in macrophages (Wright et al., 2013), but were not identified in the nasopharynx. Higher Th17 responses were found in children from Bangladesh with high carriage rates, compared to children from Sweden with lower carriage rates (Lundgren et al., 2012). In HIV^+^ individuals in Malawi where carriage rates are high, there was no evidence of a Th17 protective phenotype (Glennie et al., 2013). Furthermore, colonization by *S. pneumoniae* has been associated with decreased Th17/Treg ratios in children, possibly mediated by TGF-β induction leading to regulatory responses (Zhang et al., 2011; Neill et al., 2014; Jiang et al., 2015). Stimulation of PBMCs from individuals of different ages with three different pneumococcal proteins revealed a non-significant decrease in responses of older adults, although polyfunctionality (co-production of IFNγ by same donor) was decreased (Schmid et al., 2011). In older adults, total Th17 numbers in blood are decreased and total Treg numbers increased (van der Geest et al., 2014). Together, these observations highlight that a role of Th17 cells in conferring protection against colonization in humans remains unclear. Further investigations of mucosal Th17 and Tregs responses in older people using longitudinal studies and the EHPC model are required.

## Pneumococcal Vaccines in Older People

With the high risk of *S. pneumoniae* infection in the older population and an ageing population, there is an urgent need for effective vaccine approaches to protect this vulnerable group. The 23-valent pneumococcal polysaccharide vaccine (PPV23) is widely used in richer countries to prevent pneumococcal disease in older people and often administered alongside the influenza vaccine. Observational studies have suggested that PPV23 reduces the incidence of pneumococcal pneumonia and vaccine-serotype IPD and mortality in older individuals by 29% - 57% (Christenson et al., 2001; Andrews et al., 2004; Spindler et al., 2008; Suzuki et al., 2017). However, meta-analyses have suggested that PPV23 may only be beneficial against IPD, with no effect against the far more common non-bacteremic pneumonia (Moberley et al., 2013; Latifi-Navid et al., 2018). Along with its disputed efficacy against pneumonia, PPV23 has no protective effect against pneumococcal colonization (Adler et al., 2020).

PPV23 induces the production of anti-capsular antibodies *via* a T-cell-independent mechanism. Pneumococcal conjugate vaccines (PCV) induce higher antibody levels and longer-term immune memory *via* carrier protein mediated T-cell-dependent mechanisms. In controlled infection studies of young adults, PCV13 reduced pneumococcal colonization (Collins et al., 2015; German et al., 2019). Together with routine vaccination of children with PCV, which reduces carriage and transmission, they protect older people from *S. pneumoniae* infections through herd immunity. However, as serotype replacement threatens the efficacy of the vaccine (Lewnard and Hanage, 2019), new strategies to protect older individuals are required.

Clinical trials have confirmed that PCV13 can reduce the incidence of vaccine-serotype colonization in older adults, though the effect did not persist beyond six months (van Deursen et al., 2018). This impact of PCV13 on pneumococcal colonization may therefore be expected to overcome some of the deficiencies of PPV23 in its activity against pneumonia and, in adults >65yrs, PCV13 indeed demonstrated a 45% efficacy against non-bacteremic CAP caused by the vaccine serotypes, along with a 75% efficacy against IPD (Bonten et al., 2015). PCV20 represents a potential further advance due to the increase in serotype coverage, and results of phase 3 trials in older individuals are currently awaited (clinical trial NCT03835975) (Pfizer, 2019).

The process of inflammageing in older individuals which includes physical airway alterations, shifts in the microbiome coupled with effects on the epithelium and innate immunity, will likely contribute to a decreased efficacy of the pneumococcal vaccines. Therefore, understanding in more detail the underlying molecular and cellular mechanisms could help identify interventions to enhance immune responses in this population group. Novel vaccine approaches such as targeting pneumococcal proteins, using whole cell inactivated or attenuated strains, and with new adjuvants or immunomodulating agents to overcome the effects of inflammageing, may enhance protection against IPD in older individuals (Feldman and Anderson, 2014; Ramos-Sevillano et al., 2020; Wagner and Weinberger, 2020). Potential novel mucosal immunomodulating interventions include statins, vitamin supplementation and changes to the nasal microbiome to boost mucosal immunity, but these need to be first supported by high quality clinical trial data. In the meantime, high levels of pneumococcal vaccine uptake by adults alongside PCV vaccination of younger individuals to generate herd immunity will have the greatest effect on the morbidity and mortality associated with *S. pneumoniae* in older individuals. Additional important preventative strategies include annual health checks and smoking cessation as heart disease, diabetes mellitus and smoking increase the incidence of CAP (Torres et al., 2015).

## Discussion

The immune dysregulation associated with inflammageing has wide ranging effects on the control of pneumococcal colonization and the transition to invasive disease. To better define these processes, in parallel with murine models and *in vitro* culture systems, the EHPC model provides a unique and safe opportunity to investigate in detail the cellular and molecular changes involved. The model also enables investigation of pneumococcal-epithelial-innate immune cell interactions and activation. For example, the model can be used to assess changes in pneumococcal micro-invasion of the epithelium and characterize subsequent alterations in epithelial-derived innate immune responses following pneumococcal infection in older individuals. Transcriptomic and metabolomic approaches applied to these systems will lead to further understanding of molecular changes that occur during inflammageing and how they influence pneumococcal infection (Valdes et al., 2013; Giamarellos-Bourboulis et al., 2020).

In the last 128 years since William Osler’s observations on pneumonia, advances in the understanding of mucosal immune protection against pneumococcal disease in older individuals has progressed considerably. However, it is evident that there is still much more that needs to be discovered if we are to reduce the burden of pneumococcal disease in this vulnerable population.

## Author Contributions

CW and RH conceptualized the review. CW planned, wrote and revised the manuscript. SJ and HA wrote and revised the manuscript. JB, DF, and RH critically read and revised the manuscript. All authors contributed to the article and approved the submitted version.

## Funding

CW and RH are supported by the Medical Research Council, Grant Ref: MR/T016329/1. RH is a National Institute for Health Research (NIHR) Senior Investigator. The views expressed in this article are those of the authors and not necessarily those of the NIHR, or the Department of Health and Social Care.

## Disclaimer

The views expressed in this article are those of the authors and not necessarily those of the MRC.

## Conflict of Interest

The authors declare that the research was conducted in the absence of any commercial or financial relationships that could be construed as a potential conflict of interest.
